# Mechanisms and management of CAR T toxicity

**DOI:** 10.3389/fonc.2024.1396490

**Published:** 2024-05-21

**Authors:** Christopher J. Ferreri, Manisha Bhutani

**Affiliations:** Department of Hematologic Oncology and Blood Disorders, Levine Cancer Institute, Atrium Health Wake Forest University School of Medicine, Charlotte, NC, United States

**Keywords:** chimeric antigen receptor (CAR T), cytokine release syndrome (CRS), ICANS - immune effector cell-associated neurotoxicity syndrome, HLH - hemophagocytic lymphohistiocytosis, tocilizumab (IL-6 inhibitor), large B cell lymphoma, multiple myeloma

## Abstract

Chimeric antigen receptor (CAR) T cell therapies have dramatically improved treatment outcomes for patients with relapsed or refractory B-cell acute lymphoblastic leukemia, large B-cell lymphoma, follicular lymphoma, mantle cell lymphoma, and multiple myeloma. Despite unprecedented efficacy, treatment with CAR T cell therapies can cause a multitude of adverse effects which require monitoring and management at specialized centers and contribute to morbidity and non-relapse mortality. Such toxicities include cytokine release syndrome, immune effector cell-associated neurotoxicity syndrome, neurotoxicity distinct from ICANS, immune effector cell-associated hemophagocytic lymphohistiocytosis-like syndrome, and immune effector cell-associated hematotoxicity that can lead to prolonged cytopenias and infectious complications. This review will discuss the current understanding of the underlying pathophysiologic mechanisms and provide guidelines for the grading and management of such toxicities.

## Introduction

Chimeric antigen receptors (CAR) are engineered recombinant receptors consisting of an extracellular target antigen-binding domain, hinge region, transmembrane domain, and intracellular signaling domain that can be transduced into immune effector cells such as T cells. This allows the engineered T cells to bind the target antigen in a major histocompatibility complex (MHC)-independent fashion, followed by robust activation and expansion of the CAR T cell population which promotes tumor elimination. (1) The anti-CD19 CAR T cell therapies tisagenlecleucel (tisa-cel) and axicabtagene autoleucel (axi-cel) were the first to receive regulatory approval in 2017 after demonstrating unprecedented efficacy for the treatment of relapsed/refractory pediatric B-cell acute lymphoblastic leukemia (B-ALL) and large B-cell lymphoma (LBCL). (2–4) Subsequently, lisocabtagene maraleucel (liso-cel) received approval for relapsed/refractory LBCL, followed by both liso-cel and axi-cel becoming indicated for second-line use in LBCL based on positive phase 3 trial data. (5–7) Anti-CD19 CAR-T has additionally received regulatory approval for relapsed/refractory follicular lymphoma, adult B-ALL, and relapsed/refractory mantle cell lymphoma. (8–10) Idecabtagene vicleucel (ide-cel) and ciltacabtagene autoleucel (cilta-cel) are both B-cell maturation antigen (BCMA)-targeted CAR T cell therapies approved for the treatment of relapsed/refractory multiple myeloma after four or more prior lines of therapy (11, 12).

Despite remarkable efficacy noted with these therapies, supraphysiologic T cell activation, expansion, and systemic hyperinflammatory response mediated by cytokine production can result in potentially severe and life-threatening complications. Prototypical toxicities include cytokine release syndrome (CRS) and immune effector cell-associated neurotoxicity syndrome (ICANS). Beyond CRS and ICANS, the systemic inflammatory response triggered by CAR-T can culminate in less frequent but potentially fatal immune effector cell-associated hemophagocytic lymphohistiocytosis-like syndrome (IEC-HS). Cytopenias after CAR T cell therapy, recently termed immune effector cell-associated hematotoxicity (ICAHT), can lead to significant transfusion dependence and predispose patients to bleeding and infectious sequelae. Neurologic complications distinct from ICANS such as delayed movement and neurocognitive treatment–emergent adverse events (MNTs) are also associated with cellular therapy. (13) Post-infusion monitoring for these toxicities at specialized centers and the interventions required for toxicity management contribute significantly to the total cost of care. (14, 15) With the expected expanded indications for CAR T cell therapy in hematologic malignancies, as well as potential applications in solid tumors and autoimmune disease, optimizing prevention and treatment of the associated toxicities will become increasingly important to establish frameworks for institutions and health care providers to ultimately improve patient outcomes (16, 17).

## Cytokine release syndrome (CRS): clinical manifestations and mechanisms

The American Society for Transplantation and Cellular Therapy (ASTCT) consensus grading criteria defines cytokine release syndrome as “a supraphysiologic response following any immune therapy that results in the activation or engagement of endogenous or infused T cells and/or other immune effector cells. Symptoms can be progressive, must include fever at the onset, and may include hypotension, capillary leak (hypoxia) and end organ dysfunction.” (18)While fever is the first symptom of CRS and typically occurs within the first 14 days after infusion, clinical manifestations can range across a spectrum of severity. In less severe forms, self-limited cases may consist of fever, myalgias, fatigue, and headache. With escalating severity, CRS can lead to increased vascular permeability and capillary leak causing hypotension, pulmonary edema, or acute lung injury, requiring ICU level care. (13, 19–21) Sustained inflammation with CRS has been associated with cardiovascular toxicities of elevated serum troponin reflective of myocardial injury, decreased left ventricular ejection fraction, and cardiac arrhythmias. (22) Electrolyte derangements, liver function test abnormalities, and renal insufficiency potentially requiring temporary hemodialysis support have also been associated with CRS. (13) **Table 1** includes a summary of the incidence rate and median time to onset of CRS observed in the respective registrational clinical trial for each of the commercially available anti-CD19 and anti-BCMA CAR T cell therapies.

**Table 1:** Incidence of immune effector cell-associated toxicities seen on registrational trials for approved CAR T cell therapies.

CAR T	Indication	CRS	Neurotoxicity	Cytopenias	Infection
Tisa-cel NCT02435849 (2)	R/R B-ALL for age < 25	77% 47% G ≥ 3 Median onset 3 days	40% 13% G ≥ 3	24% with cytopenia G ≥ 3 not resolved by day 28	43% 24% G ≥ 3
Tisa-cel JULIET (3)	R/R LBCL ≥ 2 prior LOT	58% 22% G ≥ 3 Median onset 3 days	21% 12% G ≥ 3	32% with cytopenia G ≥ 3 not resolved by day 28	34% 20% G ≥ 3
Axi-cel ZUMA-1 (4)	R/R LBCL ≥ 2 prior LOT	93% 13% G ≥ 3 Median onset 2 days	64% 28% G ≥ 3	78% G ≥ 3 neutropenia 38% G ≥ 3 TCP 43% G ≥ 3 anemia	Not reported
Axi-cel ZUMA-7 (7)	Primary refractory LBCL or relapse within 12 months of 1^st^ line therapy	92% 6% G ≥ 3 Median onset 3 days	60% 21% G ≥ 3	29% with G ≥ 3 cytopenia not resolved by day 30	41% 14% G ≥ 3
Axi-cel ZUMA-5 (8)	R/R follicular lymphoma	78% 6% G ≥ 3 Median onset 4 days	56% 15% G ≥ 3	33% with G ≥ 3 cytopenia not resolved by day 30	18% G ≥ 3
Liso-cel TRANSCEND NHL 001 (5)	R/R LBCL ≥ 2 prior LOT	42% 2% G ≥ 3 Median onset 5 days	30% 10% G ≥ 3	37% with G ≥ 3 cytopenia not resolved by day 28	12% G ≥ 3
Liso-cel TRANSFORM (6)	Primary refractory LBCL, relapse within 12 months of 1^st^ line therapy, relapse and not eligible for HSCT	49% 1% G ≥ 3 Median onset 5 days	11% 4% G ≥ 3	43% with G ≥ 3 cytopenia not resolved by day 35	15% G ≥ 3
Brexu-cel ZUMA-3 (10)	Adult B-ALL	89% 24% G ≥ 3 Median onset 5 days	60% 26% G ≥ 3	36% with G ≥ 3 cytopenia not resolved by day 30	25% G ≥ 3
Brexu-cel ZUMA-2 (9)	R/R mantle cell lymphoma	91% 15% G ≥ 3 Median onset 2 days	63% 31% G ≥ 3	26% with G ≥ 3 cytopenia not resolved by day 90	32% G ≥ 3
Ide-cel KarMMa (11)	RRMM with ≥ 4 prior LOT	84% 5% G ≥ 3 Median onset 1 day	18% 3% G ≥ 3	52% with G ≥ 3 neutropenia and 62% with G ≥ 3 TCP not resolved by day 28	22% G ≥ 3
Cilta-cel CARTITUDE-1 (12)	RRMM with ≥ 4 prior LOT	95% 5% G ≥ 3 Median onset 7 days	17% ICANS 2% G ≥ 3 12% other neurotoxicities, 9% G ≥ 3	30% with G ≥ 3 neutropenia and 41% with G ≥ 3 TCP not resolved by day 30	20% G ≥ 3

CRS, cytokine release syndrome; R/R, relapsed/refractory; B-ALL, B-cell acute lymphoblastic leukemia; G, grade; LBCL, large B-cell lymphoma; LOT, lines of therapy; IEC-HS), immune effector cell-associated hemophagocytic lymphohistiocytosis-like syndrome; TCP, thrombocytopenia; HSCT, hematopoietic stem cell transplantation; RRMM, relapsed/refractory multiple myeloma.

Inflammatory markers and cytokines that have been implicated in CRS include tumor necrosis factor-alpha (TNF-α), interferon-gamma (IFN-ɣ), interleukin-1 (IL-1), IL-2, soluble IL2Rα, IL-4, IL-6, IL-8, IL-10, ferritin, C-reactive protein (CRP), granulocyte/macrophage colony stimulating factor (GM-CSF), macrophage inflammatory protein-1α (MIP-1α), monocyte chemoattractant protein-1 (MCP-1), granzyme B, and soluble gp130. (23) The term “cytokine release syndrome” was first used in the early 1990s after systemic reactions associated with elevations in TNF-α and IFN-ɣ were noted after immunosuppressive treatment with the anti-T-cell antibody muromonab-CD3 (OKT3), which was tempered with glucocorticoids. Subsequently, CRS has been associated with various immunotherapies including monoclonal antibodies, antibody-drug conjugates, immune checkpoint inhibitors, bispecific T cell redirecting antibodies, and CAR T cell therapies. (24) Similar elevations in serum cytokines were observed in the first clinical studies of CAR T cell therapies for chronic lymphocytic leukemia (CLL) and B-ALL (19, 25, 26).

Activation of the vascular endothelium has also been implicated in the pathophysiology of CRS. RNA *in-situ* hybridization studies from a patient who died due to CRS after treatment with anti-CD19 CAR T cell therapy noted that both interstitial cells and vascular endothelium lining cells expressed IL-6, which was markedly elevated. (27) Patients with grade ≥2 CRS after anti CD19 CAR T infusion had increased prothrombin time (PT), activated partial thromboplastin time (aPTT), fibrinogen, D-dimer, factor VIII, von Willebrand factor (vWF), and decreased platelet count and antithrombin levels compared to those with lower grade or no CRS, suggesting that endothelial activation was predictive for greater CRS severity and development of disseminated intravascular coagulopathy (DIC). (28) A multivariable analysis of 133 adult patients treated with anti-CD19 CAR T noted that biomarkers of endothelial activation including angiopoietin-2 and vWF were increased during severe CRS, and also relatively increased prior to lymphodepletion chemotherapy in patients who subsequently developed CRS. Severe thrombocytopenia prior to lymphodepletion was predictive for development of CRS, and it was postulated that these patients were more susceptible to endothelial activation due to platelets being a primary source of the endothelial stabilizing cytokine angiopoietin-1 (29).

While retrospective analysis of serum cytokines in relation to CRS severity have been informative in understanding the underlying pathophysiology, the development of animal models of cytokine release syndrome has also been critical to further elucidating these mechanisms. Experiments from a murine model of B-ALL treated with anti-CD19 CAR T demonstrated that CAR T cells produced negligible levels of IL-6, but instead bystander proinflammatory monocytes and macrophages were responsible for IL-1 and IL-6 production. It was noted that IL-1 secretion preceded IL-6 production by several hours, and treatment with the IL-1 receptor inhibitor anakinra was equally effective as the IL-6 receptor inhibitor tocilizumab in treating CRS in this model. (30) Another murine model of CRS developing after anti-CD19 CAR T cell therapy for B-ALL also demonstrated that CRS severity was mediated by IL-6, IL-1, and nitric oxide production from recipient macrophages rather than directly from the CAR T cells themselves (31).

Though IL-1 and IL-6 secretion primarily occurs from bystander monocytes and macrophages in CRS, upstream IFN-ɣ secretion occurs from activated CAR T cells and hence IFN-ɣ production is used as a potency assay for CAR T cell effector function. IFN-ɣ activates innate immune cells such as macrophages to upregulate antigen-presentation pathways, which also leads to immune checkpoint expression. *In vitro* experiments utilizing xenograft models of hematologic malignancy and serum samples obtained from patients who developed clinically significant CRS demonstrated that IFN-ɣ inhibition and deletion resulted in decreased macrophage activation and proinflammatory cytokine production, as well as reduced immune checkpoint expression. Compared to inhibition of IL-1 and IL-6, IFN-ɣ blockade dampened downstream proinflammatory cytokine production to a greater extent. (32) In a simplified humanized murine model of CRS/neurotoxicity after treatment with anti-CD19 CAR T, treatment with the IFN-ɣ inhibitor emapalumab led to decreased inflammatory signaling and toxicity mitigation without impacting CAR T efficacy. (33) However, studies from a fully immunocompetent mouse model demonstrated that treatment with IFN-ɣ knockout CAR T cells resulted in similar levels of proinflammatory cytokine elevation and occurrence of clinical CRS in the treated mice compared to those treated with the wild-type CAR T. While possible that IFN-ɣ signaling plays a more significant role in humans compared to mice, these results suggest that *in vivo* IFN-ɣ signaling may be redundant or not directly causal for CRS development (34).

Lastly, an *in vitro* model of the macrophage-endothelial interface suggested that endothelial inflammation occurs after CAR T cell therapy independent of signaling from macrophages, which in turn amplifies macrophage-mediated inflammatory signaling. The transcription factor STAT3 was implicated in hyperinflammatory signaling driven by the activated endothelium, and inhibition of the JAK-STAT pathway with ruxolitinib in combination with dexamethasone resulted in the greatest reduction of pro-inflammatory cytokine secretion. (35) Further refinement of *in vivo* animal models will be pivotal for discovering the more precise molecular underpinnings of CRS development and severity.

## Factors associated with CRS risk

Identifying factors associated with an increased risk for the development of severe CRS can help inform decisions about ensuing monitoring and management. Such factors may be intrinsic to the CAR product or related to patient and disease specific factors. The dose of infused CAR T cells has been shown to be one such intrinsic factor, with several studies noting that CRS was observed more commonly in patients treated with a higher cell dose. (29, 36). Analysis of clinical trials and real-world experience studies have suggested that treatment with CARs incorporating a CD28 costimulatory motif may pose a greater risk for incidence and severity of CRS compared to those with a 4–1BB costimulatory domain. (21, 36, 37) Several studies have suggested that CD4-postiive CAR T cells contribute to CRS development to a greater degree than CD8-positive CAR T cells, indicating that the CD4/CD8 ratio of the CAR product may be potentially be predictive for CRS (34, 38).

Regarding disease and patient specific factors, a higher disease burden prior to CAR T cell infusion (e.g., degree of bone marrow involvement prior to lymphodepletion) has generally been associated with increased severity of CRS. (21, 39–42) The underlying disease biology also impacts CRS risk, with patients having a more proliferative malignancy such as B-ALL or LBCL seemingly at risk for higher grade CRS compared to those with more indolent disease such as follicular lymphoma or multiple myeloma. (8, 11, 12, 43–45) The modified Endothelial Activation and Stress Index (m-EASIX) score consists of laboratory parameters that are readily available (lactate dehydrogenase [LDH in U/L] x C-reactive protein [CRP in mg/dL]/platelets [PLTs in 10^9^ cells/L]). In a retrospective analysis of 118 patients receiving anti-CD19 CAR T cell therapy for the treatment of either B-ALL or LBCL, the m-EASIX score prior to infusion was associated with onset of any grade and severe CRS, and m-EASIX score on days +1 and +3 post-infusion were associated with onset of severe CRS. Furthermore, when analyzing individual variables (CRP, LDH, PLTs, creatinine), lower platelet counts and higher CRP were independently associated with the onset of severe CRS. Thus, the m-EASIX score can be used as a predictor of endothelial activation which may indicate increased risk for more severe CRS (46).

## Grading and management of CRS

There were initially multiple heterogeneous definition and grading systems for CRS when CAR T cell therapies were first being studied in humans, such as the Common Terminology Criteria for Adverse Events (CTCAE), University of Pennsylvania, Memorial Sloan Kettering Cancer Center (MSKCC), and CAR-T cell therapy-associated toxicity (CARTOX) criteria. (41, 47–49) A multi-institutional effort to refine CRS grading, known as the Lee criteria, defined fever as a hallmark of CRS and included hypotension responsive to a low dose of one vasopressor as grade 2 CRS. Furthermore, the Lee criteria incorporated a treatment algorithm that corresponded with each grade of toxicity. (50) Given the differences among the published grading systems for CAR T-associated toxicity, an expert panel was convened to draft the ASTCT consensus grading criteria for CRS and neurologic toxicity associated with immune effector cells in 2018. (18) The ASTCT consensus criteria have since been applied as a uniform grading system for subsequent clinical trials and real-world analyses.

Given that symptoms of CRS are non-specific, the symptoms defining CRS must be attributed specifically to treatment with the immune effector cell therapy. In addition to having a reasonable temporal relation, typically with onset occurring within 14 days of therapy, other causes of fever, hypotension, or hypoxia such as sepsis should be excluded. While there is overlap between CRS and other immune effector cell-associated toxicities (ICANS, IEC-HS), these toxicities are excluded from the definition of CRS and are graded separately. Laboratory parameters of inflammation such as CRP and ferritin are often trended for patients receiving cell therapy; however, specific laboratory parameters are excluded from the definition and grading of CRS due to the non-specificity of these markers of inflammation, and because cytokine panels are not readily available at most institutions. For the purposes of defining CRS, fever is defined by the CTCAE version 5.0 criteria as a temperature ≥ 38.0 °C. The severity of CRS is defined by the degree of hypotension and hypoxia, as other specific organ toxicities generally occur in the setting of hypotension and hypoxia and do not influence treatment decisions with glucocorticoids or anti-cytokine therapy. While fever may resolve quickly after the initiation of anti-cytokine therapies, CRS is not considered resolved until all signs and symptoms leading to the original diagnosis of CRS have resolved unless they can be clearly attributed to an alternative etiology. The ASTCT CRS consensus grading system is summarized in **Table 2** (18).

**Table 2:** The ASTCT consensus grading criteria for cytokine release syndrome (CRS).

CRS Parameter	Grade 1	Grade 2	Grade 3	Grade 4
Fever*	Temperature ≥ 38.0 °C at onset for all grades			
With				
**Hypotension**	None	Not requiring vasopressors	Requiring a vasopressor with or without vasopressin	Requiring multiple vasopressors (excluding vasopressin)
And/or†				
**Hypoxia**	None	Requiring low-flow nasal cannula‡ or blow-by	Requiring high-flow nasal cannula‡, facemask, nonrebreather mask, or Venturi mask	Requiring positive pressure (e.g., CPAP, BiPAP, intubation and mechanical ventilation)

*Fever is defined as temperature ≥ 38.0 °C not attributable to another cause. In patients who have CRS then receive antipyretic or anti-cytokine therapy, fever is no longer required to grade subsequent CRS severity. In this case CRS grading is driven by hypotension and/or hypoxia.

†CRS grade is determined by the more severe event if both hypotension and hypoxia are present.

‡Low-flow nasal cannula is defined as oxygen delivered at ≤ 6 liters per minute. High-flow nasal cannula is defined as oxygen delivered at ≥ 6 liters per minute.

This table was adapted from the ASTCT Consensus Grading for Cytokine Release Syndrome and Neurologic Toxicity Associated with Immune Effector Cells. (18)

Acknowledging the relatively high incidence of CRS with most approved CAR T cell therapies, patients require close monitoring initially with frequent vital sign assessment, laboratory studies (complete blood count, complete metabolic panel, coagulation studies, inflammatory markers), and neurologic assessment. (13) Outpatient administration has been successfully demonstrated in both clinical trial and real-world settings; however, the majority of CAR T cell therapies administered to date have occurred inpatient with a defined period of monitoring for the development of the associated toxicities. (51–53) Management of CRS involves evaluating for infectious etiologies of fever, supportive care, and varying degrees of immunosuppressive therapy based on CRS severity. (13) Tocilizumab is a monoclonal antibody antagonist of the IL-6 receptor that has received regulatory approval for the treatment of CRS. (54) Each approved CAR T cell therapy is administered through a Risk Evaluation and Mitigation Strategy (REMS) program which mandates tocilizumab availability at the site of treatment in case it is needed for the management of CRS. Furthermore, the prescribing information for each CAR product includes management guidance for each grade of CRS (55–60).

As fever without hypotension or hypoxia defines grade 1 CRS, initial management consists of excluding infectious etiology with blood cultures, urine culture, and chest radiography. The underlying hematologic malignancy and lymphodepletion chemotherapy administered prior to CAR T often result in neutropenia concomitant with onset of fever, and thus neutropenic fever should be managed with broad spectrum antibiotics and granulocyte stimulating factor (G-CSF) per institutional guidelines. Grade 1 CRS can often be managed with supportive care alone, such as antipyretics, intravenous fluids, and symptomatic management; however, patients with recurrent or persistent fever may be managed with tocilizumab as per grade ≥ 2 CRS (61).

Management of grade 2 CRS includes intravenous fluids for hypotension and/or supplemental oxygenation as needed. Patients with grade ≥2 CRS should undergo more intensive monitoring with continuous pulse oximetry and cardiac telemetry. All patients with grade 2 CRS should receive tocilizumab 8 mg/kg IV (dose capped at 800 mg/dose). Tocilizumab may be given every eight hours for a maximum of four total doses. For patients with persistent borderline hypotension after reasonable intravenous fluid bolus administration and one to two doses of tocilizumab, the addition of glucocorticoid therapy with dexamethasone 10 mg IV (or equivalent) every 12 hours should be considered. If brisk improvement is not observed, vasopressor therapy should be initiated for grade 3 CRS and level of care should be escalated to an intensive care unit. The management of grade 3 CRS includes supportive care for hypotension and hypoxia with vasopressors and escalating supplemental oxygenation as necessary, and echocardiography should be performed to assess cardiac function. Tocilizumab and glucocorticoid should be administered concurrently, with tocilizumab dosing as per labeled use above and with glucocorticoid dose increased to dexamethasone 10 mg IV every 6 hours (or equivalent) followed by a rapid taper once symptoms are noted to improve. Refractory grade 3 or escalation to grade 4 CRS may necessitate further titration of glucocorticoid dose up to a maximum methylprednisolone dose of 1,000 mg IV every 12 hours plus consideration of alternative anti-cytokine or immunosuppressive therapies after tocilizumab has been administered for the maximum of four doses (61).

The management of CRS refractory to standard anti-inflammatory therapy with tocilizumab and glucocorticoids can pose significant challenges, with data often limited to case reports or retrospective series. It is first prudent to exclude active infection or progression of malignancy as a contributing factor. (62) As murine models of CAR T-associated toxicities have implied IL-1 as an important pathophysiologic mediator of both CRS and ICANS, the IL-1 receptor antagonist anakinra, which is approved for the treatment of rheumatoid arthritis and neonatal-onset multisystem inflammatory disease, has become increasingly used for the treatment of refractory toxicity. (30, 31, 62) While anakinra has demonstrated greater efficacy in the treatment of refractory ICANS, it has been shown to facilitate resolution of refractory CRS in case reports and retrospective studies (63, 64).

Siltuximab is a monoclonal antibody that binds directly to IL-6 preventing its interaction with IL-6 receptors, whereas tocilizumab is an inhibitor of the IL-6 receptor. Siltuximab has been demonstrated to be efficacious in the treatment of CRS both alone and in combination with tocilizumab. (5, 65, 66) The TNF-α receptor inhibitor etanercept, approved for various rheumatologic indications, has been shown to abate CRS in case reports of both anti-CD19 and anti-BCMA CAR T cell therapies. (26, 67) Emapalumab is a monoclonal antibody inhibitor of IFN-ɣ approved for the treatment of primary hemophagocytic lymphohistiocytosis (HLH). Preclinical studies have supported a potential role for its use in CAR T-associated toxicities, and emapalumab was given with success for a case of refractory grade 4 CRS with secondary HLH. (33, 68) Furthermore, in a case series of pediatric patients with B-ALL who had refractory CRS despite IL-6 inhibition, glucocorticoids, and IL-1 blockade, a single dose of emapalumab 1 mg/kg resulted in a profound decrease in inflammatory markers and significant clinical improvement in five of six patients treated (69).

The tyrosine kinase inhibitor dasatinib has been demonstrated to inhibit the lymphocyte-specific protein tyrosine kinase, which impairs downstream signaling in various CAR constructs leading to a cessation in cytokine production, cytolytic activity, and in decreased CAR T cell proliferation in both *in vitro* and *in vivo* models. In a murine CRS model, dasatinib was able to mitigate the effects of CRS and CAR-T cells were able to regain antitumor cytolytic activity after dasatinib discontinuation. (70, 71) In one published case of grade 3 CRS and grade 4 ICANS observed after anti-CD19 CAR T cell therapy for LBCL despite treatment with four doses of tocilizumab and high-dose glucocorticoids, treatment with dasatinib 100 mg daily for seven days resulted in substantial clinical improvement. While CAR T expansion was noted to decline markedly for this patient after dasatinib initiation without evidence of re-expansion after discontinuation, it did not appear to hamper efficacy as the patient was in an ongoing complete remission over two years from the time of CAR T cell infusion. (72)

Inflammatory cytokines such as IFN-ɣ, IL-6, IL-12, and TNF-α signal through Janus kinase 1 (JAK1), with activation of JAK leading to phosphorylation of signal transducer and activator of transcription proteins (STATs) which subsequently modulates gene expression. The selective JAK1 inhibitor itacitinib was able to effectively reduce CRS-related cytokines produced by anti-CD19 CAR T cells in a dose-dependent fashion in both *in vitro* and *in vivo* models without impacting CAR T proliferation or cytolytic function. (73) This preclinical rationale for JAK-STAT inhibition has been translated to the clinical setting with several case reports noting resolution of refractory CRS after treatment with the JAK-inhibitor ruxolitinib. (74–77) Lastly, a variety of different inducible safety switches to either reversibly or irreversibly impair CAR T cell function have been designed as a mechanism for potentially managing severe toxicities; however, they have yet to become significant in clinical practice. (78) Pharmacotherapy for the management of CRS is summarized in **Table 3**.

**Table 3:** Pharmacotherapy for the treatment of cytokine release syndrome.

CRS Grade	Pharmacotherapy Recommendations
Grade 1	• Broad-spectrum antibiotics if concomitant neutropenia • Anti-pyretics (acetaminophen) • Consider Tocilizumab for persistent or refractory cases
Grade 2	• Intravenous fluids for hypotension and/or supplemental oxygen • Tocilizumab 8 mg/kg IV may be given every eight hours for a maximum of four total doses • Consider adjunctive dexamethasone 10 mg IV every 12 hours for persistent or refractory cases
Grade 3 or Grade 4	• Vasopressors for hypotension and/or supplemental oxygen • Tocilizumab 8 mg/kg IV may be given every eight hours for a maximum of four total doses • Adjunctive dexamethasone 10 mg IV every 6 hours (or equivalent), which can be escalated up to a dose of methylprednisolone 1,000 mg IV every 12 hours for refractory cases • Consider alternative anti-cytokine or immunosuppressive therapies for refractory cases after tocilizumab
Alternative Therapies for Refractory CRS	
**Anakinra –** IL-1 receptor antagonist, case reports and retrospective studies demonstrating efficacy (63, 64)	
**Siltuximab –** IL-6 inhibitor, demonstrated efficacy (5, 65, 66)	
**Etanercept** – TNF-α receptor inhibitor, demonstrated efficacy in case reports (26, 67)	
**Emapalumab** – IFN-ɣ inhibitor, demonstrated efficacy in case report (69)	
**Dasatinib** – tyrosine kinase inhibitor, demonstrated efficacy in case report (72)	
**JAK-STAT inhibitors** • Itacitinib has been shown to reduce levels of CRS-related cytokines in pre-clinical studies (73), and has been shown to reduce grade ≥ 2 CRS when used as prophylaxis prior to axi-cel (79) • Ruxolitinib has demonstrated efficacy in several case reports (74–77)	

CRS, cytokine release syndrome; IV, intravenous; mg, milligrams; kg, kilogram; IL, interleukin; TNF, tumor necrosis factor; IFN, interferon.

Because the management of CRS often requires pharmacologic intervention with anti-cytokine therapy or glucocorticoids, there has been theoretical concern that such anti-inflammatory therapy may hamper the efficacy of CAR T cell therapy. Preclinical studies did not suggest hindrance of antitumor activity with IL-1, IL-6 and IFN-ɣ inhibition respectively. (30, 33) A retrospective analysis regarding the timing of tocilizumab administration for patients treated with anti-BCMA CAR T cell therapy demonstrated that patients receiving tocilizumab earlier (<12 hours from CRS onset) experienced a shorter median duration of CRS without negative effect on response or median progression-free survival outcomes. (80) There have been conflicting reports regarding corticosteroid therapy, with one retrospective study demonstrating no impact of corticosteroid therapy on response rate and CAR T expansion/persistence in B-ALL patients, whereas another retrospective analysis in LBCL patients suggested that a higher cumulative dose and prolonged use of corticosteroids had a negative impact on progression-free survival. (81, 82) Ultimately, randomized studies with long-term follow up will be required to discern whether certain toxicity management strategies have a positive or negative effect on CAR T cell therapy efficacy.

There has also been investigation into whether prophylactic or preemptive approaches to toxicity management can decrease the incidence and severity of both CRS and ICANS. Such attempts include early intervention with tocilizumab or corticosteroids for lower grade toxicity, as well as prophylaxis strategies with corticosteroids, tocilizumab, anakinra, or the JAK1 inhibitor itacitinib. (79, 83–90) Results from studies investigating prophylactic interventions are summarized in **Table 4**.

**Table 4:** Prophylactic approaches for mitigation of CRS/ICANS in patients treated with anti-CD19 CAR T.

Agent/Study	Outcomes	Comparator	Comments
**Dexamethasone** 10 mg on days 0, 1, and 2 ZUMA-1, cohort 6 (88)	CRS 80% G ≥ 3 CRS 0% ICANS 58% G ≥ 3 ICANS 13%	ZUMA-1, cohorts 1–2 (no prophylactic dex): CRS 93%, G ≥ 3 13% ICANS 64%, G ≥ 3 28%	Lower baseline tumor burden in prophylactic dex cohort compared to cohorts 1–2
**Tocilizumab** on day 2 after axi-cel infusion Safety expansion cohort of ZUMA-1 (87)	G ≥ 3 CRS 3% G ≥ 3 ICANS 35%	ZUMA-1, cohorts 1–2 (no prophylactic toci): G ≥ 3 CRS 13% G ≥ 3 ICANS 28%	Peak IL-6 levels were higher in prophylactic toci cohort
**Anakinra** on days 0–7 NCT04432506 (91)	CRS 95% G ≥ 2 CRS 40% G ≥ 3 CRS 5% ICANS 35% G ≥ 3 ICANS 20%	Tumor-burden matched retrospective cohort: G ≥ 2 CRS 50% G ≥ 3 ICANS 30%	No observed impact on expansion kinetics or CAR T efficacy
**Anakinra** on day 2 through at least day 10 post-CAR T NCT04148430 (92)	CRS 74% G ≥ 3 CRS 6.4% ICANS 19% G ≥ 3 ICANS 9.7%	No comparison cohort	Favorable reduction in all grades of ICANS compared to historical controls
**Itacitinib** (JAK1 inhibitor) starting day -3 through day +26 after CAR T NCT04071366 (79)	CRS 65% Grade 2 CRS 17% G ≥ 3 CRS 0% ICANS 13% G ≥ 2 ICANS 9%	Placebo-controlled cohort: CRS 87% Grade 2 CRS 57% G ≥ 3 CRS 0% ICANS 35% G ≥ 2 ICANS 22%	Tocilizumab use lower in itacitinib arm (17% v. 57%). Persistent G ≥ 3 neutropenia and TCP at day 28 higher in itacitinib arm
**Defibrotide** on days -5 to -3 pre-CAR T and days 0–7 post-CAR T; NCT03954106 (93)	ICANS 50% G ≥ 3 ICANS 25%	No comparison cohort	Study terminated early as unlikely to meet 1° endpoint
**Lenzilumab** (GM-CSF inhibitor) 6 hours prior to axi-cel ZUMA-19 (94)	G ≥ 3 CRS 0% G ≥ 3 ICANS 17%	No comparison cohort	Met 1° endpoint for safety, but terminated after only 6 patients

CRS, cytokine release syndrome; ICANS, immune effector cell-associated neurotoxicity syndrome; G, grade; dex, dexamethasone; toci, tocilizumab; TCP, thrombocytopenia; 1°, primary; GM-CSF, granulocyte-macrophage colony-stimulating factor.

## Immune effector cell-associated neurotoxicity syndrome (ICANS): clinical manifestations and mechanisms

The ASTCT consensus grading criteria defines ICANS as “a disorder characterized by a pathologic process involving the central nervous system following any immune therapy that results in the activation or engagement of endogenous or infused T cells and/or other immune effector cells. Symptoms or signs can be progressive and may include aphasia, altered level of consciousness, impairment of cognitive skills, motor weakness, seizures, and cerebral edema.” Other forms of neurotoxicity potentially attributable to CAR T cell therapies such as headache, tremor, myoclonus, and asterixis are not considered as ICANS-defining, are graded separately by NCI-CTCAE criteria, and are managed symptomatically. (18) ICANS has been noted to occur concurrently with CRS, shortly after CRS subsides, in the absence of CRS, and in some instances with delayed onset up to one month after infusion. It often first manifests with tremor, mild expressive aphasia, impaired attention, dysgraphia, apraxia, and mild lethargy. Expressive aphasia appears to be the most common and specific symptom of ICANS. At higher grades of severity, ICANS may result in refractory seizures, necessitate intubation for airway protection, and can rarely result in fatal cerebral edema (18, 61).

Observations from imaging of the central nervous system in patients with ICANS has generally observed that findings are symmetric and have a propensity to affect uniquely susceptible brain regions. Involvement of the thalami and deep gray matter indicate that ICANS is likely prompted by a systemic process such as the inflammation mediated by inflammatory cytokines. Specific imaging findings such as leptomeningeal enhancement and T2 hyperintensity of the cerebral sulci, T2 hyperintensity and swelling of the bilateral thalami, and T2 hyperintensities in the supratentorial white matter can bear resemblance to injury patterns seen in other infectious or inflammatory central nervous system (CNS) conditions. Elevation of cerebrospinal fluid (CSF) protein on lumbar puncture samples has been commonly observed in patients with ICANS, and elevated cytokine levels in the CSF typically mirror cytokine elevations observed in serum samples. (95) Electroencephalogram (EEG) findings in patients with ICANS have most commonly been notable for generalized periodic discharges generated from both cortical and subcortical regions which may be induced by proinflammatory cytokines or directly mediated by T cells (96).

Elevated serum and CSF cytokines have been implicated in the pathophysiology of ICANS. Despite the strong association with CRS and inflammatory cytokines, the pathophysiology remains poorly understood in that ICANS can occur without antecedent CRS, and patients with severe CRS do not always develop ICANS. Based on serum and CSF cytokine analyses pooled from multiple human studies, IFN-ɣ, IL-15, IL-6, IL-10, GM-CSF, and IL-1RA appear to be most strongly associated with ICANS. Additionally, IL-2, IL-2Rα, CXCL10, and Granzyme b seem to be possibly associated with ICANS. (95) In a retrospective analysis of 53 patients treated with anti-CD19 CAR T for B-ALL, higher serum elevations of the proinflammatory cytokines IL1α, IL2, IL3, IL5, IL6, IL10, IL15, INF-γ, interferon-gamma inducible protein-10 (IP10), G-CSF, GM-CSF, and MCP1 by day three after CAR T was associated with severe neurotoxicity. Those with severe neurotoxicity were observed to have disproportionately high CSF levels of IL6, IL8, MCP1, and IP10, potentially indicating localized CNS production rather than just mirroring serum cytokine elevations (97).

Studies from animal models have also supported cytokine elevations in association with neurotoxicity. In the murine model of CRS and ICANS which demonstrated that IL-1 and IL-6 were primarily derived from activated monocytes and macrophages, treatment with tocilizumab did not abate symptoms of neurotoxicity when given at the onset of fever, whereas anakinra was able to improve neurotoxicity severity and survival. Furthermore, prophylactic tocilizumab did not protect the mice from delayed lethal neurotoxicity, whereas prophylactic anakinra prevented both CRS and neurotoxicity development. These murine studies suggested that IL-1 may be more integral to the pathophysiology of ICANS and further supported investigation of IL-1 inhibition for ICANS treatment and prevention in subsequent human studies. (30) In a rhesus macaque model of neurotoxicity after adoptive transfer of anti-CD20 CAR T cells, neurotoxicity was associated with serum elevations in IL-8, IL-8, IL-1RA, CXCL9, and CXCL11, and with disproportionately elevated CSF levels of IL-6, IL-2, GM-CSF, and VEGF levels. Pan-T cell encephalitis was observed in the brain parenchyma of the non-human primates experiencing neurotoxicity as evidenced by the accumulation of both CAR T and non-CAR T cells (98).

As seen with CRS, evidence from multiple studies have demonstrated endothelial activation and disruption as a mechanism underlying the pathophysiology of ICANS. Endothelial activation resulting from systemic cytokine release after CAR T cell therapy may predispose to the CNS microvascular dysfunction noted in ICANS. Perturbations in the angiopoietin (Ang)-Tie 2 axis have been associated with ICANS, as Ang-2 is released from endothelial cells upon activation from proinflammatory cytokines. Ang-1 binding to the Tie-2 receptor typically facilitates vascular quiescence and integrity; however, Ang-2 displaces Ang-1 from its receptor and induces vascular permeability. (95) In two separate retrospective analyses of patients treated with CAR T for B-ALL, patients who developed severe neurotoxicity were more likely to have laboratory findings consistent with DIC and capillary leak, lowers levels of Ang-1, higher levels of Ang-2, and a higher Ang-2:Ang-1 ratio suggestive of endothelial activation. (97, 99) Another retrospective study found that ICANS occurrence was associated with an increased PT, D-dimer, Factor VII level, and vWF level, and with decreased serum levels of fibrinogen and platelet count. (28) CSF samples from patients experiencing ICANS compared to baseline were notable for elevated protein concentrations, leukocyte counts (including CAR T cells), and serum cytokines reflective of increased blood-brain barrier permeability. When human brain vascular pericytes were exposed *in vitro* to IFN-ɣ and TNF-α, pericyte secretion of IL-6 and VEGF was observed which further increased blood-brain barrier disruption. An autopsy specimen from a patient who developed fatal neurotoxicity after anti-CD19 CAR T was notable for platelet aggregation, vWF binding in small capillaries, and multifocal vascular disruption with multifocal hemorrhages indicative of endothelial activation (99).

A subset of patients with ICANS develop excitatory neurotoxicity manifested by seizures. Glutamate and quinolinic acid are endogenous excitatory agonists of the N-methyl-D-aspartate (NMDA) receptor. Analysis of CSF from 13 patients with pre-treatment samples who then developed excitatory neurotoxicity revealed that both glutamate and quinolinic acid were significantly elevated during the neurotoxicity period compared to baseline. On the contrary, these endogenous NMDA agonists were not elevated in a patient without ICANS who underwent pre-treatment and day 14 lumbar punctures. These findings indicate that endothelial activation and increased blood-brain barrier permeability contributing to CSF cytokine elevation may be linked to elevations of these excitatory agonists in the CNS. (97) It has been proposed that neurotoxicity may reflect an on-target, off-tumor effect of anti-CD19 CAR T cell therapy given that CD19 expression was observed by single-cell RNA sequencing analysis of human brain mural cells, which support the vasculature. However, this appears to be an unlikely mechanism given that ICANS has subsequently been observed with CAR T therapies targeting alternative antigens such as BCMA and GPRC5D (11, 12, 100).

## Factors associated with ICANS risk

Risk factors for ICANS intrinsic to the CAR T product identified from several patient cohorts include higher CAR T dose, higher peak CAR T expansion, and early/higher elevations of serum proinflammatory cytokines likely related to CAR T expansion kinetics. (97, 99) Additionally, the incorporation of a CD28 costimulatory domain as in axi-cel and brexu-cel appears to be associated with higher incidence and severity of ICANS compared to the other approved products that incorporate a 4–1BB costimulatory domain (**Table 1**) (4, 7, 9, 10).

In terms of patient and disease-specific factors, a high burden of disease prior to lymphodepletion has been associated with increased ICANS risk. While ICANS can occur in the absence of CRS, developing early and severe CRS after CAR T infusion has been associated with increased incidence and severity of ICANS. (97, 99) One analysis identified pre-existing neurologic comorbidities as a risk factor for subsequent ICANS development. (99) Another study observed that thrombocytopenia was significantly associated with an increased risk for grade ≥ 3 neurotoxicity. (101) The modified EASIX score at day three after CAR T infusion was found to be associated with onset of severe ICANS, though the pre-infusion m-EASIX score was not associated with the development of ICANS. Analyzing the individual components of the m-EASIX score, higher CRP and lower platelet count were both associated with ensuing onset of severe ICANS (46).

## Grading and management of ICANS

While ICANS has become the preferred term for this specific manifestation of neurotoxicity due to immune effector cell therapies, the registrational trials for the approved CAR products were done at a time when the NCI-CTCAE criteria for neurotoxicity were being utilized and thus there is more heterogeneity in the way neurotoxicity has been defined, graded, and reported in the literature. (18, 55–60) The CARTOX criteria provided the first step toward streamlining the objective grading of neurotoxicity with the incorporation of a screening tool called the CARTOX-10, which included a ten point scale to assess the patient for alterations in speech, orientation, concentration, and handwriting. The complete CARTOX neurotoxicity grading system involved evaluation of level of consciousness, motor symptoms, seizures, and for signs of elevated intracranial pressure (ICP) with CSF opening pressure measurement and papilledema grading, which can be challenging to obtain and objectively quantify. (49) The ASTCT consensus criteria incorporates a modified version of the CARTOX-10 screening tool called the Immune Effector Cell-Associated Encephalopathy (ICE) score (**Table 5**), and simplifies assessment of the other domains to facilitate more objective ICANS grading (**Table 6**) (18).

**Table 5:** The immune effector cell-associated encephalopathy (ICE) score.

**Orientation**	Orientation to year, month, city, hospital: 4 points (1 point each)
**Naming**	Name 3 objects (e.g., clock, pen, button): 3 points
**Following Commands**	Ability to follow simple commands (e.g., Show me 2 fingers or close your eyes and stick out your tongue): 1 point
**Writing**	Ability to write a standard sentence (e.g., Our national bird is the bald eagle): 1 point
**Attention**	Count backwards from 100 by 10: 1 point

This table was adapted from the ASTCT Consensus Grading for Cytokine Release Syndrome and Neurologic Toxicity Associated with Immune Effector Cells. (18)

10: No impairment.

7–9: Grade 1 ICANS.

3–6: Grade 2 ICANS.

0–2: Grade 3 ICANS.

0 due to patient unarousable and unable to perform ICE assessment: Grade 4 ICANS.

**Table 6:** ASTCT consensus grading for ICANS in adult patients.

Neurotoxicity Domain	Grade 1	Grade 2	Grade 3	Grade 4
ICE score*	7–9	3–6	0–2	0 (patient is unarousable and unable to perform ICE
**Depressed level of consciousness**†	Awakens spontaneously	Awakens to voice	Awakens only to tactile stimulus	Patient is unarousable or requires vigorous or repetitive tactile stimuli to arouse. Stupor or coma
**Seizure**	N/A	N/A	Any clinical seizure focal or generalized that resolves rapidly or nonconvulsive seizures on EEG that resolve with intervention	Life-threatening prolonged seizure (>5 min); or repetitive clinical or electrical seizures without return to baseline in between
**Motor findings**‡	N/A	N/A	N/A	Deep focal motor weakness such as hemiparesis or paraparesis
**Elevated ICP/cerebral edema**	N/A	N/A	Focal/local edema on neuroimaging§	Diffuse cerebral edema on neuroimaging; decerebrate or decorticate posturing; or cranial nerve VI palsy; or papilledema; or Cushing’s triad

ICANS grade is determined by the most severe event not attributable to any other cause.

*A patient with an ICE score of 0 may be classified as grade 3 ICANS if awake with global aphasia, but a patient with an ICE score of 0 may be classified as grade 4 ICANS if unarousable.

†Depressed level of consciousness should be attributable to no other cause (e.g., no sedating medication).

‡Tremors and myoclonus associated with immune effector cell therapies may be graded according to CTCAE v5.0, but they do not influence ICANS grading.

§Intracranial hemorrhage with or without associated edema is not considered a neurotoxicity feature and is excluded from ICANS grading. It may be graded according to CTCAE v5.0.

This table was adapted from the ASTCT Consensus Grading for Cytokine Release Syndrome and Neurologic Toxicity Associated with Immune Effector Cells. (18)

N/A, not applicable.

**Table 7:** Pharmacotherapy for the treatment of ICANS.

ICANS Grade	Pharmacotherapy Recommendations
Grade 1	• Supportive care, consider dexamethasone 10 mg and reassess
Grade 2	• Dexamethasone 10 mg IV every 12 hours, can escalate dosing to 10 mg every 6 hours for persistent grade 2 ICANS • Continue corticosteroids until improvement to grade 1 ICANS, then rapidly taper as clinically appropriate
Grade 3 or Grade 4	• Dexamethasone 10 mg IV every 6 hours • Can escalate up to methylprednisolone 1,000 mg IV given two to three times daily for refractory grade 3 or grade 4 ICANS • Seizures and/or status epilepticus should be managed with anti-epileptics with neurology assistance as per institutional guidelines • Alternative therapies should be considered for the treatment of ICANS refractory to corticosteroids
Alternative Therapies for Refractory ICANS	
**Anakinra –** IL-1 receptor antagonist, multiple retrospective studies demonstrating efficacy (64, 102, 103)	
**Siltuximab –** IL-6 inhibitor, pre-clinical rationale without significant clinical demonstration of efficacy. Note that tocilizumab (IL-6R inhibitor) should only be used for concomitant CRS as inhibition of the receptor causes transient increases in free IL-6 which may exacerbate ICANS (65, 97, 104)	
**Dasatinib** – tyrosine kinase inhibitor, demonstrated efficacy in case report (72)	
**Intrathecal hydrocortisone and/or chemotherapy** – direct targeting of CAR T cells in CSF, demonstrated efficacy in case reports and retrospective studies (105, 106)	
**Antithymocyte globulin (ATG) –** direct targeting of CAR T cells, demonstrated efficacy when used as part of multimodal therapy in case report (104)	
**Cyclophosphamide** – Chemotherapeutic targeting of CAR T cells, demonstrated efficacy in case report (107)	

ICANS, immune effector cell-associated neurotoxicity syndrome; IV, intravenous; IL, interleukin; CRS, cytokine release syndrome; CSF, cerebrospinal fluid.

The incidence of neurotoxicity observed with each approved CAR T product in their respective registrational trials is summarized in **Table 1**; however, it is important to note that the ASTCT consensus criteria were not yet implemented for these trials. The ASTCT consensus grading criteria for ICANS is outlined in **Table 6**. The overall ICANS grade is dictated by the most severe grade observed across the five neurotoxicity domains (ICE score, depressed level of consciousness, seizure, motor findings, and elevated ICP/cerebral edema). Grade 1 ICANS is defined by an ICE score in the range of 7–9 and the patient must be able to awaken spontaneously. An ICE score of 3–6 constitutes grade 2 ICANS, with the patient being able to awaken to voice. Any clinical or electrical seizure that resolves rapidly with intervention or focal areas of edema on neuroimaging qualify as grade 3 ICANS, as does an ICE score ranging 0–2 if patient awakens only to tactile stimulus. A patient with an ICE score of 0 who is unarousable and unable to perform the ICE questionnaire is defined as having grade 4 ICANS. Other qualifiers for grade 4 ICANS include life-threatening prolonged seizures or repetitive clinical or electrical without interim return to baseline, deep focal motor weakness, diffuse cerebral edema on neuroimaging, decerebrate or decorticate posturing, cranial nerve VI palsy, papilledema, and Cushing’s triad. Patients with grade 4 ICANS often require intubation and mechanical ventilation for airway protection and seizure management, but this should not also be classified as grade 4 CRS if the need for mechanical ventilation is not secondary to refractory hypoxia (18).

Pharmacotherapy for the management of ICANS is summarized in **Table 7**. The prescribing information for each approved CAR T includes guidance for the management of neurologic toxicities. (55–60) After CAR T infusion, patients should be monitored closely with documentation of the ICE score and assessment of motor strength at least twice daily. Patients should have serial laboratory monitoring (CBC, CMP, coagulation studies, inflammatory markers) and severe hyponatremia should be corrected. Initiating anti-epileptic therapy for seizure prophylaxis is common at most institutions. Neurology consultation should be sought after noted onset of ICANS, and grade ≥ 2 neurotoxicity should prompt further evaluation with neuroimaging and EEG. Lumbar puncture for CSF analysis and opening pressure can be considered for patients with grade 2 ICANS and strongly encouraged for those with grade ≥ 3 ICANS. Tocilizumab may be administered for situations where CRS is concurrent with ICANS, but in the absence of CRS the mainstay of ICANS treatment is supportive care and corticosteroid therapy (61).

Patients with grade 1 ICANS can often be monitored and managed with supportive care alone, but it is reasonable to treat with dexamethasone 10 mg and reassess. Increasingly severe ICANS is generally managed with increased corticosteroid dosing, such as dexamethasone 10 mg every 12 hours for grade 2 ICANS, and escalation to 10 mg every 6 hours for persistent grade 2 or development of grade 3 ICANS. Corticosteroids should be continued until improvement to grade 1 ICANS and then rapidly tapered as deemed clinically appropriate. Patients with grade ≥ 3 ICANS should be monitored in an ICU setting. Seizures and status epilepticus should be managed with neurology expertise per institutional guidelines. With persistent grade 3 ICANS or emergence of grade 4 toxicity, corticosteroid dose can be further increased to 1,000 mg methylprednisolone given two to three times daily. Alternative therapies should be considered for management of ICANS refractory to corticosteroids, which may include anakinra, ruxolitinib, siltuximab, cyclophosphamide, antithymocyte globulin, or intrathecal hydrocortisone with or without chemotherapy. (61) As with CRS, attempts to prevent or mitigate the severity of ICANS prior to its onset are being investigated. **Table 4** summarizes recent investigations into such prophylactic approaches.

There is a lack of high-quality evidence to inform the management of ICANS refractory to corticosteroid therapy, and it can be challenging to isolate the effect of a particular intervention in the setting of patients receiving simultaneous or overlapping therapies. With preclinical studies suggesting a fundamental role for IL-1 in the pathogenesis of ICANS, anakinra has become a preferred agent for the management of refractory ICANS. (30) Clinical responses with ICANS improvement or resolution have been reported in retrospective analyses from multiple institutions, though timing of initiation, dosing, and concurrent therapies have been heterogeneous. (64, 102, 103) A recent retrospective analysis across nine institutions identified 40 patients treated with anakinra for grade ≥ 2 ICANS without improvement on corticosteroid therapy, which was demonstrated to be well tolerated. Treatment with a high-dose anakinra regimen (defined as >200 mg/day IV) was associated with improved clinical outcomes compared to a lower dose (100–200 mg/day), as evidenced by 0% treatment-related mortality in the high-dose group compared to 47% in the low-dose group, and a median cumulative incidence of CRS/ICANS resolution from the time of anakinra initiation of seven days in the high-dose group compared to median time to resolution not being reached in the low-dose due to the higher rate of treatment-related mortality (64).

The IL-6 receptor inhibitor tocilizumab has not been shown to be effective in the treatment of ICANS. Due to its inhibition of IL-6R rather than IL-6 itself, transient increases in serum and CSF IL-6 have been documented after tocilizumab initiation and it has been hypothesized that this may exacerbate ICANS as tocilizumab is not thought to penetrate the blood-brain barrier well. For example, in 16 patients with severe neurotoxicity who received tocilizumab, 56% had their peak neurotoxicity grade occur after the first dose of tocilizumab administration. (97) Whereas siltuximab is an antagonist of IL-6, there is some rationale that treatment with this agent would be advantageous for ICANS outcomes compared to tocilizumab. (65) However, clinical experience with siltuximab specifically for the treatment of refractory ICANS has not been well documented. (65, 104) The tyrosine kinase inhibitor dasatinib has demonstrated an ability to impair CAR T proliferation and downstream cytokine secretion in both *in vitro* and *in vivo* models, supporting a rationale for clinical use. (70, 71) In one patient who received dasatinib for refractory grade 4 ICANS with concurrent grade 3 CRS, treatment with dasatinib led to a rapid improvement in neurologic function and allowed for extubation one day later (72).

In addition to interfering with cytokine signaling, direct targeting of CAR T cells has been explored for the management of refractory ICANS. A case report of two patients treated with intrathecal hydrocortisone plus chemotherapy for corticosteroid-refractory grade 3 and 4 ICANS respectively led to substantial clinical improvement. (105) A retrospective analysis noted that for seven patients who received early intrathecal hydrocortisone with or without intrathecal chemotherapy within five days of developing high-grade ICANS, all seven patients had subsequent resolution of ICANS. Of the four patients with refractory high-grade ICANS who did not receive intrathecal therapy or received it late (>5 days after onset), only two patients recovered from ICANS. Additionally, the median duration of steroid use and cumulative steroid dose were lower for the patients receiving early intrathecal therapy. (106) In one case of refractory grade 4 ICANS with cerebral edema, three doses of rabbit antithymocyte globulin (ATG) were given as part of the multimodal therapy with a temporal suggestion of benefit as the patient was extubated five days after starting ATG and ICANS had resolved completely within two weeks. (104) Another case described a patient who developed refractory rebound neurotoxicity characterized by the development of brain MRI findings consistent with posterior reversible encephalopathy syndrome (PRES) which coincided with a continued rise to peak circulating CAR T cell levels in the peripheral blood. The patient was treated with cyclophosphamide 1.5 g/m2 to target the CAR T cells and significant clinical improvement was noted over the subsequent days and with resolution of MRI abnormalities four weeks later (107).

## Other neurotoxicities distinct from ICANS

Neurotoxicity distinct from ICANS has also been observed after CAR T cell therapies. Of particular concern are the described movement and neurocognitive treatment-emergent adverse events (MNTs) that have been described after administration of anti-BCMA CAR T. These defined MNTs consist of a potential range of symptoms falling into one of three categories: movement disorder (e.g., micrographia, resting tremor), cognitive impairment (e.g., memory loss, disturbance in attention), or personality changes (e.g., reduced facial expression, tremors). For patients treated with cilta-cel on the CARTITUDE-1 study, patients were defined as having MNTs if they reported symptoms in at least two of these categories. Symptoms must have occurred after recovery from CRS and/or ICANS and felt to be attributable to the CAR T cell therapy. Using these criteria, five patients (5%) developed MNTs after cilta-cel with median onset of 27 days post-infusion, and a median onset of 17 days (range, 3–94) after resolution from CRS/ICANS. These toxicities were generally not responsive to corticosteroids or other supportive measures, which included anakinra, siltuximab, systemic and intrathecal chemotherapy, dasatinib, and carbidopa/levodopa for parkinsonism. One patient’s death was attributed to such neurotoxicity, and only one patient’s MNTs were resolving at data cutoff. Patients who experienced MNTs were retrospectively determined to have at least two of the following features: high tumor burden, grade ≥ 2 CRS or any grade ICANS, and high CAR T expansion/persistence in peripheral blood. After mitigation strategies were implemented, which included more aggressive bridging therapy to reduce disease burden prior to cilta-cel infusion, early intervention for CRS/ICANS, handwriting assessments for early detection, and an extended monitoring period for neurotoxicity, incidence of MNTs has been noted to be < 1% for patients treated with cilta-cel on subsequent trials. (108)

While the clinical manifestations of MNTs seen after anti-BCMA CAR T can have a presentation similar to Parkinson disease, autopsy results from two of the patients who developed such toxicity after cilta-cel suggest an alternative pathophysiology. Both patients were noted to have normal pigmentation in the substantia nigra and symptoms did not improve with a trial of carbidopa/levodopa. There was notable focal gliosis and T cell infiltrate (CD8 > CD4) in the basal ganglia. It was not determined whether these were CAR-positive T cells, however these patients did have persistent CAR T elevations in the blood and CSF. (108, 109)One patient was noted to have detectable BCMA expression on neurons and astrocytes in the caudate nucleus of the basal ganglia, as well as in neurons of the adjacent frontal cortex. (109) This autopsy finding coupled with RNA sequencing analysis of healthy donors observing low levels of BCMA RNA expression in the basal ganglia suggest a possible on-target, off-tumor mechanism for this form of neurotoxicity. (110) While more commonly observed after cilta-cel, MNTs manifesting as parkinsonism have also been observed after treatment with ide-cel. (59) Clinical management of MNTs remains challenging, as they have generally not been responsive to corticosteroids or anti-inflammatory therapies. One case report demonstrated that treatment with cyclophosphamide 2 grams/m^2^ aimed at significantly reducing the persistent CAR T cell population resulted in resolution of refractory parkinsonism symptoms. (111)

In addition to ICANS and MNTs, other neurotoxicities observed after anti-BCMA CAR T cell therapy include peripheral sensory and motor neuropathies, ataxia, nystagmus, facial nerve paralysis and other cranial nerve palsies, diplopia, and concentration impairment. (108) CAR T cell therapy targeting the G protein-coupled receptor, class C, group 5, member D (GPRC5D) are under clinical investigation for the treatment of multiple myeloma with promising preliminary results. At the highest dose-level evaluated, two patients developed dose-limiting toxicities of cerebellar toxicity characterized by wide-based gait, saccadic eye movements, appendicular and truncal ataxia, and dysarthria. Treatment with steroids and intravenous immune globulin (IVIG) resulted in stabilization of symptoms but without resolution. Given that microarray data from six healthy donors noted that GPRC5D expression was most enriched in the olivary nucleus, it is possible that this represents on-target, off-tumor toxicity. (100) Other rare distinct neurotoxicities observed after CAR T cell therapy include transverse myelitis/myelopathy, human herpesvirus 6-associated myelitis, acute leukoencephalopathy, central diabetes insipidus, Guillain-Barre-like syndrome, persistent postural tremors resembling essential tremor, new onset migraine headaches, and various peripheral neuropathies (112–117).

## Immune effector cell-associated hemophagocytic lymphohistiocytosis-like syndrome (IEC-HS): clinical manifestations, risk factors, and mechanisms

Secondary hemophagocytic lymphohistiocytosis (HLH) can occur after CAR T due to overactivation of the immune system. Typically characterized by hyperferritinemia, coagulopathy, hepatic dysfunction and cytopenias among other HLH-like manifestations, this is a lesser known but potentially fatal complication of CAR T that has become increasingly recognized. Consequently, US Food and Drug Administration package inserts incorporate the black box warning risk of HLH for both commercially approved BCMA -targeted CAR T cell therapies. (59, 60) The chronology and symptomatology of HLH-like toxicities can be difficult to distinguish from severe CRS. The earliest of the reports described laboratory features mimicking HLH/MAS superimposed on severe CRS in a 7-year-old girl with recurrent B-ALL who was treated with CD19 CAR T cell therapy. (26) Subsequent reports have demonstrated that HLH-like syndrome can occur as a late manifestation when signs of conventional CRS appear to be resolving. (118, 119) Recognizing the need to better delineate HLH-like toxicities following CAR T and to provide a framework for cross-trial comparisons, an ASTCT expert panel recently named this complication as IEC-HS, which is defined as “the development of a pathological and biochemical hyperinflammatory syndrome independent from CRS and ICANS that (1) manifests with features of MAS/HLH (2), is attributable to immune effector cell therapy, and (3) is associated with progression or new onset of cytopenias, hyperferritinemia, coagulopathy with hypofibrinogenemia, and/or transaminitis.” (120)

Clinical suspicion and early recognition of IEC-HS is critical. CRS in moderate to severe forms may manifest with overlapping features of IEC-HS (e.g., elevated ferritin, elevated transaminases, hemophagocytosis, coagulopathy, cytopenia, and multi-organ dysfunction) with indistinguishable cytokine and proteomic profiles. (18, 118, 121) Given significant overlap, it is critically important to distinguish between IEC-HS and prolonged severe or recurrent CRS as treatment implications differ between the two. Timing of IEC-HS in relation to antecedent CRS can vary, but it is a distinct manifestation independent from CRS that occurs once CRS is resolved/resolving. While CRS typically develops and resolves within 2 weeks of CAR-T administration, IEC-HS arises after CRS and may persist for weeks. The consensus panel strongly cautions against using IEC-HS nomenclature to describe patients with severe CRS involving multiorgan dysfunction. Malignancy progression, underlying rheumatologic/metabolic disorders, severe infections, or other etiologies that can trigger secondary HLH should be excluded (120).

The HLH-2004 criteria and the H score, the common scoring systems used for diagnosis of secondary HLH, have low validity and applicability in identifying IEC-HS as most patients treated with CAR T have alternative explanations for traditional criteria and thresholds used for diagnosing HLH (e.g., elevated ferritin, fever and cytopenias). (122–124) In 2017, a CARTOX Working Group was formed that proposed a diagnosis of HLH should be made if the ferritin levels peak at >10,000 ng/mL within the CRS window of 5 days after CAR T-cell therapy, and if the patient develops any two of the following: grade 3 or greater organ toxicities involving the liver, kidneys, or lungs, or hemophagocytosis in the bone marrow or other organs (49). Considering vast heterogeneity in baseline ferritin values among patients undergoing CAR T, waiting to reach an arbitrarily set threshold for ferritin elevation could cause unnecessary delays in diagnosis of IEC-HS, therefore the ASTCT working group has not incorporated specific ferritin values in their proposed diagnostic criteria of IEC-HS. Although there is no specific cutoff, a substantial elevation (defined as >2 times the upper limit of normal or baseline at time of infusion) or rapidly rising serum ferritin is a prerequisite for the diagnosis of IEC-HS. Acknowledging that not all patients with antecedent or ongoing CRS will progress to IEC-HS, the ASTCT working group considers the timing as a key factor in diagnosing IEC-HS. While CRS develops within 14 days post-infusion, IEC-HS is often of delayed onset and is associated with progression or new onset laboratory abnormalities or clinical deterioration as CRS is resolved/resolving. Common manifestations of IEC-HS include hepatic transaminase elevation, hypofibrinogenemia, other coagulation abnormalities, hemophagocytes in bone marrow or other tissue, cytopenias (new onset, worsening, or refractory), elevated LDH, direct hyperbilirubinemia, and new onset splenomegaly. Other manifestations that may be present include renal insufficiency, pulmonary complications, neurotoxicity, fever, and hypertriglyceridemia (120).

Risk factors that have been implicated in the development of IEC-HS include baseline disease burden, CAR T proliferation dynamics, immune resistance mechanisms, target antigen, CAR T cell construct, costimulatory domain, T cell selection, cell dose and patient characteristics (baseline inflammation, immune suppression, cytopenias, genetic predisposition). (45, 118, 119, 125, 126). While the underlying pathophysiology of IEC-HS remains to be fully elucidated, similarities to CRS have been observed. The sustained T cell activation via engineered CAR recognition of tumor antigen results in a hyperinflammatory response, T cell activation and proliferation, cytokine release, and resultant macrophage activation and release of soluble factors creating a positive feedback loop. (120) Like primary and secondary HLH, where defects in cytolytic function of NK cells and CTLs can lead to pathologic T-cell expansion, it has been postulated that the profound and persistent NK cell lymphopenia in concert with heightened CAR T cell expansion and delayed T cell contraction can predispose select patients with CRS to develop a secondary hyperinflammatory response in form of IEC-HS. (118) In the context of 59 patients infused with CD22 CAR T cells where a substantial proportion developed IEC-HS, disproportionately low absolute number of NK cells relative to CD8 T cells in peripheral blood pre-infusion, and higher bone marrow T cell to NK cell ratio was noted in patients who later developed IEC-HS. (118) In line with this observation, a recent murine model of HLH demonstrated that both CTL and cytotoxic NK cells contribute to the development of HLH-like syndrome in mice after infection with lymphocytic choriomeningitis virus (127).

Certain cytokines (e.g., IFN-γ, IL-6, IL-10, IL-18, sIL-2R, CXCL9), and proteomic profiles overlap between HLH and severe CRS. (118, 121). Unlike in CRS, IL-1 appears to be a central player in IEC-HS. IL-1β levels were particularly high among those patients with IEC-HS, which supports the use of anakinra, an IL-1 receptor antagonist, for management of this syndrome. (118) IFN-ɣ, a mediator of systemic inflammation and macrophage activation, is thought to play a key role in development of IEC-HS, which is supported by the high serum level observed in a patient who developed IEC-HS post tisa-cel and was treated with emapalumab with rapid defervescence and improvement in clinical and laboratory parameters (128).

## Grading and management of IEC-HS

The ASTCT expert panel developed a grading schema for IEC-HS formed based on the NCI-CTCAE category “Immune system disorder, other.” Grades of IEC-HS range from 1–5 with grade 1 toxicity representative of asymptomatic or mild symptoms not requiring intervention, and grade 5 reflecting death due to IEC-HS. Toxicity warranting intervention is labeled as grade 2, with grade 3 toxicity being severe but not immediately life-threatening, and grade 4 IEC-HS is considered life-threatening (e.g., life-threatening bleeding or hypotension, respiratory distress requiring intubation, dialysis indicated for acute kidney injury) (120).

The ASTCT expert panel has proposed a stepwise approach to the treatment of IEC-HS. Therapy should be initiated prior to the development of life-threatening complications. The use of tocilizumab or siltuximab in patients with IEC-HS without CRS is discouraged. First line therapy with the IL-1 receptor antagonist anakinra, with or without corticosteroids, is recommended given its potential to disrupt secondary HLH/MAS associated with rheumatologic and other disorders. Anakinra has a short half-life allowing rapid titration to effect, a well-established side effect profile, and pharmacokinetic data to support both intravenous and subcutaneous use. (120) Anakinra has been observed to be efficacious in IEC-HS in patients treated with CD19 CAR T cell therapy for pediatric B-ALL, DLBCL, and mantle cell lymphoma. (129, 130) Anakinra can be given at a dose of 100–200 mg every 6–12 hours in adults, or up to 10 mg/kg IV daily. If there is no improvement within 48 hours despite escalation of anakinra to target dose with concurrent corticosteroids, initiation of the JAK1/2 inhibitor ruxolitinib as second line therapy is recommended. Ruxolitinib, via inhibition of JAK/STAT signaling pathways, can reduce secretion of the pro-inflammatory cytokines implicated in the development of IEC-HS such as IL-2, IL-6, and IFN-γ. (120) Case reports and retrospective studies have demonstrated its clinical efficacy for primary and secondary HLH (131, 132).

For severe IEC-HS refractory to anakinra and ruxolitinib, additional agents like the anti-IFN-γ monoclonal antibody emapalumab or low-dose etoposide chemotherapy can be considered. Treatment with emapalumab may be more appropriate in the context of significantly elevated serum IFN-γ, whereas etoposide to deplete T cells may be more applicable for a documented persistent elevation of CAR T cells. (120) Emapalumab binds both free and receptor-bound IFN-γ, and elevated levels of each have been documented in both primary HLH and CAR-T associated toxicities. (133, 134) It is FDA-approved for primary HLH in children and adults in the setting of refractory, recurrent or progressive disease or intolerance to other standard treatments. The initial dosing is a twice-weekly intravenous infusion of 1 mg/kg. Evidence pertaining to its efficacy in IEC-HS is limited to case reports describing the clinical recovery of patients with severe IEC-HS and CRS refractory to corticosteroids and multiple anti-cytokine treatments who received emapalumab as salvage therapy. (68, 128) The topoisomerase inhibitor etoposide is cytotoxic to T cells and has been shown to have efficacy in primary and secondary HLH in protocols such as HLH-94. (135, 136) The suggested dosing in adults is 50–100 mg/m2 once to twice weekly. (137) Antithymocyte globulin (ATG) and alemtuzumab have been proposed as salvage therapy options in primary pediatric HLH, but their use for IEC-HS is not well established (120).

Patients with IEC-HS require frequent clinical assessment, daily laboratory studies (CBC, CMP, coagulation studies, inflammatory markers), and diligent supportive care. Such supportive care includes transfusion support for cytopenias, cryoprecipitate for hypofibrinogenemia, vitamin K and/or fresh frozen plasma for severe coagulopathy, and infectious disease consultation for both diagnostic purposes and infection prophylaxis recommendations given prolonged immunosuppression. As clinical and laboratory parameters stabilize, tapering immunosuppression is encouraged while monitoring for recrudescence of symptoms. Further retrospective and prospective studies are necessary to characterize and differentiate severe CRS complicated by HLH-like manifestations versus IEC-HS, and to inform optimal monitoring and therapeutic strategies (120).

## Immune effector cell-associated hematotoxicity (ICAHT): clinical manifestations, risk factors, and mechanisms

Anemia, thrombocytopenia, and leukopenia with neutropenia are among the most common toxicities observed after treatment with CAR T cell therapy. The incidence of cytopenias observed with each approved CAR T cell therapy on the respective registrational study is summarized in **Table 1**, noting the limitations of comparisons among trials. In a pooled real-world analysis of 235 patients treated with anti-CD19 CAR T, incidence of grade ≥3 neutropenia, anemia, and thrombocytopenia were 91%, 69%, and 62% respectively. While early cytopenias can be attributed to the effects of lymphodepletion chemotherapy prior to CAR T, persistent or late recurrence of cytopenias is likely multifactorial in nature and can result in significant transfusion dependence, bleeding complications, and infectious sequelae (138–140).

Three distinct clinical phenotypes of neutrophil recovery have been observed after CAR T. The most common recovery pattern observed in a multicenter retrospective analysis was that of intermittent recovery in 52%, consisting of initial recovery with G-CSF stimulation for the neutropenia following lymphodepletion followed by a biphasic second reduction in absolute neutrophil count (ANC) in month two post-CAR T, and then with another recovery in the subsequent weeks. A subset of 25% demonstrated quick recovery from the initial neutropenia, whereas 23% of patients displayed an aplastic phenotype characterized by a protracted course of neutropenia despite G-CSF use (139).

A multitude of risk factors have been associated with the development of cytopenias after CAR T cell therapy. Such risk factors include malignancy-related features (e.g., underlying disease and disease burden), number and type of prior therapies, baseline bone marrow characteristics (e.g., pre-existing cytopenias, clonal hematopoiesis of indeterminate potential [CHIP]), inflammatory status prior to CAR T, and factors intrinsic to the CAR T product. Additionally, post-infusion toxicities such as CRS, IEC-HS, or infection influence the risk of developing prolonged cytopenias. (140) Calculated prior to lymphodepletion (day −5), the CAR-HEMATOTOX score incorporates both factors that relate to the baseline inflammatory state (e.g., CRP, ferritin) and the patient’s hematopoietic reserve (e.g., hemoglobin, ANC, platelet count). This model was first developed to predict severe hematotoxicity in relapsed/refractory LBCL and was then subsequently extended to patients receiving CAR-T therapy for mantle cell lymphoma and multiple myeloma. High-risk patients with score ≥2 had a longer duration of neutropenia, higher incidence of severe thrombocytopenia and anemia, increased rates of severe infection, higher non-relapse mortality, and inferior treatment outcomes compared to their low-risk counterparts (score 0–1) (139–142).

There are several factors to consider regarding the pathophysiology of ICAHT. Baseline cytopenias prior to CAR T treatment likely reflect underlying impairment of the hematopoietic stem and progenitor cell compartment secondary to prior cytotoxic therapies. The presence of preexisting CHIP may contribute to an underlying inflammatory state and contribute to the subsequent development of prolonged cytopenias. (143, 144) Patients with cytopenia prior to CAR T are more likely to have increased bone marrow disease burden, which in turn is correlated with a longer time to hematologic recovery after CAR T. Patients with delayed or prolonged cytopenias after anti-BCMA CAR T were shown to have a higher percentage of persistent CAR T cells in the bone marrow aspirate two months after infusion, suggesting that CAR T-mediated inflammation may contribute to these cytopenias. (145) This is further supported by the observation that patients developing hyperinflammatory complications such as CRS and IEC-HS are more likely to develop prolonged cytopenias. (146, 147) In patients with an aplastic phenotype, single cell sequencing studies have shown an inflammatory micromilieu in the form of oligoclonal CAR-T-cell expansion, T-cell receptor restriction, clonally expanded CXCR1^hi^, and IFN-γ expressing cytotoxic T cells (148, 149) Additionally, reactivation of certain viruses (human herpesvirus 6, cytomegalovirus, Epstein-Barr virus, adenovirus etc.) in the setting of immune suppression can directly suppress hematopoietic cell recovery (140).

## Grading and management of ICAHT

The NCI-CTCAE criteria have been used for grading the hematologic adverse events in the respective registrational CAR T studies. With the goal of more accurately representing the neutropenia observed after T-cell-based immunotherapies, the European Hematology Association (EHA) and European Society for Blood and Marrow Transplantation (EBMT) formed an expert panel that established the terminology and grading system for ICAHT. Early ICAHT is defined as occurring within the first 30 days after CAR T, and late ICAHT refers to cytopenia observed after day 30. The grading system (grade 1–4) incorporates both depth and duration of neutropenia for early ICAHT, whereas late ICAHT grading is reflective of the depth of neutropenia (140).

The EHA/EBMT expert panel recommends the use of the CAR-HEMATOTOX score to identify patients at elevated risk for developing severe neutropenia and continued use of current grading systems for anemia and thrombocytopenia. A tiered approach to diagnostic evaluation has been proposed for persistent cytopenia. Initial workup includes ruling out common infectious etiologies, vitamin deficiencies, contribution from medications, and persistent inflammatory etiologies (CRS/IEC-HS). For those with sustained ICAHT, additional evaluation to rule out less common infectious etiologies and bone marrow aspiration and biopsy to investigate underlying bone marrow disease is warranted (140).

Management of ICAHT consists primarily of transfusion support, growth factor administration, and infectious prophylaxis. Administration of G-CSF has been demonstrated to shorten the duration of severe neutropenia and reduce infectious complications. (140) Retrospective analyses from real-word data sets have demonstrated faster neutrophil recovery and an acceptable safety profile with early G-CSF without increases in the rate of high-grade CRS or ICANS. (150–152) Notably, G-CSF did not impact CAR-T expansion or efficacy. (153) Most patients will adequately respond to growth factor support with count recovery. (146) While data for patients treated with CAR T remains limited, thrombopoietin (TPO) agonists have been given successfully to patients with prolonged or late thrombocytopenia, and have also been noted to improve anemia in some instances. (140) B-cell aplasia and hypogammaglobulinemia associated with CAR T can compound the risk of infections, therefore IgG replacement should be considered for those with high infection risk or experiencing recurrent infections with a recommended goal IgG trough > 400 mg/dL. (154) For patients with persistent ICAHT refractory to the aforementioned interventions, hematopoietic stem cell boost with cryopreserved autologous CD34^+^ stem cells has been observed to result in improvement or resolution of neutropenia in a majority of cases (155–157).

## Secondary malignancies observed after CAR T cell therapy

The US Food and Drug Administration (FDA) released a statement on November 28, 2023, regarding the potential risk of secondary T cell malignancies in patients treated with CAR T cell therapy, which include CAR-positive T cell lymphoma. There have been 20 cases of T cell malignancies reported after CAR T cell infusion in approximately 8,000 cases conveyed to the FDA Adverse Events Reporting System. With an estimated total number of commercial CAR T infusions greater than 30,000, the suggested incidence of secondary T cell malignancies is quite low, but further studies are needed to better define the true incidence and to further elucidate the pathogenesis. Furthermore, the Centers for International Blood and Marrow Transplant Research (CIBMTR) database has documented 565 cases of secondary or subsequent malignancies in 485 individual patients out of a total of 8,060 enrolled in post-authorization safety studies. Such malignancies are most commonly non-melanomatous skin cancers and therapy-related myelodysplastic syndrome (MDS) or acute myeloid leukemia (AML). (158) For example, 10 of the 97 patients who received cilta-cel on the CARTITUDE-1 study have since been noted to have secondary myeloid malignancies. (60, 159) As such, the FDA has stated that patients who have received CAR T cell therapy should be monitored indefinitely for the development of subsequent malignancies but that benefits of CAR T in treating the malignancy at hand continues to outweigh such risks. (158) It is particularly challenging to determine the causal association of CAR T with the development of secondary malignancy given the existing confounders related to prior systemic therapies received (including high-dose alkylating chemotherapy for autologous stem cell transplant), patient age, and immortal time bias. Long-term follow up of patients enrolled to studies with CAR T in earlier line treatment settings may help to elucidate this further (159).

## Conclusion

In conclusion, CAR T cell therapy has revolutionized the treatment of hematologic malignancies and efforts are ongoing to extend the benefits of cellular therapies to patients with solid tumors and rheumatologic disease. Despite remarkable efficacy, the development of potentially severe immune effector cell-associated toxicities require that patients receive close monitoring at centers with expertise in the diagnosis and management of these adverse effects. Significant progress has been made in the understanding of the underlying pathophysiology of such toxicities by means of retrospective analyses and animal models, which have informed therapeutic approaches to toxicity management. The development of consensus guidelines for the definition and grading of these distinct toxicities will further facilitate future cross-trial comparisons and prospective studies, which will be critical for optimizing risk-stratification and management approaches. For the future, active areas of investigation include the optimization of preclinical models to better elucidate the mechanisms of CAR T toxicities, identification of predictive biomarkers, prospective trials of prophylactic or preemptive toxicity mitigation, and creative CAR designs to further ameliorate the development of such toxicities.

## Author contributions

CF: Conceptualization, Writing – original draft, Writing – review & editing. MB: Conceptualization, Writing – original draft, Writing – review & editing.
